# Mortality after inpatient treatment for diarrhea in children: a cohort study

**DOI:** 10.1186/s12916-019-1258-0

**Published:** 2019-01-28

**Authors:** Alison Talbert, Moses Ngari, Evasius Bauni, Martha Mwangome, Neema Mturi, Mark Otiende, Kathryn Maitland, Judd Walson, James A. Berkley

**Affiliations:** 1 KEMRI/Wellcome Trust Research Programme, Centre for Geographic Medicine Research - Coast, PO Box 230, Kilifi, 80108 Kenya; 2 The Childhood Acute Illness & Nutrition Network (CHAIN), Nairobi, Kenya; 3 Wellcome Trust Centre for Clinical Tropical Medicine, Imperial College, London, UK; 4 Department of Global Health, University of Washington, Seattle, USA; 5 Center for Tropical Medicine & Global Health, University of Oxford, Oxford, UK

**Keywords:** Diarrhea, Children, Mortality, Inpatient, Post-discharge, Africa, Kenya

## Abstract

**Background:**

There is an increasing recognition that children remain at elevated risk of death following discharge from health facilities in resource-poor settings. Diarrhea has previously been highlighted as a risk factor for post-discharge mortality.

**Methods:**

A retrospective cohort study was conducted to estimate the incidence and demographic, clinical, and biochemical features associated with inpatient and 1-year post-discharge mortality amongst children aged 2–59 months admitted with diarrhea from 2007 to 2015 at Kilifi County Hospital and who were residents of Kilifi Health and Demographic Surveillance System (KHDSS). Log-binomial regression was used to identify risk factors for inpatient mortality. Time at risk was from the date of discharge to the date of death, out-migration, or 365 days later. Post-discharge mortality rate was computed per 1000 child-years of observation, and Cox proportion regression used to identify risk factors for mortality.

**Results:**

Two thousand six hundred twenty-six child KHDSS residents were admitted with diarrhea, median age 13 (IQR 8–21) months, of which 415 (16%) were severely malnourished and 130 (5.0%) had a positive HIV test. One hundred twenty-one (4.6%) died in the hospital, and of 2505 children discharged alive, 49 (2.1%) died after discharge: 21.4 (95% CI 16.1–28.3) deaths per 1000 child-years. Admission with signs of both diarrhea and severe pneumonia or severe pneumonia alone had a higher risk of both inpatient and post-discharge mortality than admission for diarrhea alone. There was no significant difference in inpatient and post-discharge mortality between children admitted with diarrhea alone and those with other diagnoses excluding severe pneumonia. HIV, low mid-upper arm circumference (MUAC), and bacteremia were associated with both inpatient and post-discharge mortality. Signs of circulatory impairment, sepsis, and abnormal electrolytes were associated with inpatient but not post-discharge mortality. Prior admission and lower chest wall indrawing were associated with post-discharge mortality but not inpatient mortality. Age, stuntedness, and persistent or bloody diarrhea were not associated with mortality before or after discharge.

**Conclusions:**

Our results accentuate the need for research to improve the uptake and outcomes of services for malnutrition and HIV as well as to elucidate causal pathways and test interventions to mitigate these risks.

**Electronic supplementary material:**

The online version of this article (10.1186/s12916-019-1258-0) contains supplementary material, which is available to authorized users.

## Introduction

Children admitted to a hospital, successfully treated, and discharged home are at higher risk of dying in the subsequent period compared to healthy children in the community [1]. Diarrheal disease is a common cause of pediatric admission and is reported to be associated with increased risk of post-discharge mortality in several settings in resource-poor countries [1–3]. Although sub-Saharan Africa has the highest incidence of diarrheal disease, few studies have examined the mortality following treatment for diarrhea [4].

A systematic review of pediatric post-discharge mortality in resource-poor countries in 2013 identified only three studies specifically evaluating mortality following diarrhea and all were from Bangladesh [2, 3, 5, 6]. These studies, conducted between 1979 and 1992 in both urban and rural hospitals, followed a total of 1329 children for 12 weeks to 1 year after hospital discharge and reported 71 deaths. The studies reported inconsistent associations between malnutrition and risk of post-discharge death and did not evaluate the impact of HIV on post-discharge mortality. This review also identified three papers examining diarrhea as one of several risk factors for mortality after general pediatric admissions: two from Kenya and one from Guinea Bissau [7–9]. Only one study analyzed the independent effects of nutritional and HIV status on outcome [8]. Since that review (up to October 2012), 17 additional studies reporting outcomes of treatment of children with diarrhea in low- and middle-income countries have been published up to the end of May 2018. However, none reported on post-discharge mortality (Additional file 1).

The Global Enteric Multicenter Study (GEMS) examined the etiology of moderate to severe diarrhea in 9439 children aged 0 to 59 months in seven countries from Africa and Asia which reported that 2.0% of children with diarrhea died within 90 days post-enrolment, representing a markedly increased risk of death (OR 8.5 (95% CI 5.8–12.5)) compared to 13,129 age-matched community children without diarrhea [10]. Two thirds of these diarrhea-associated deaths occurred more than 7 days after enrolment, and mortality was highest in children less than 2 years old. This study did not disaggregate deaths that occurred during hospitalization and after discharge.

In this study, we aimed to estimate inpatient and 1-year post-discharge mortality after admission for diarrheal disease in children aged 2 months to 5 years in rural Kenya and to describe risk factors for an increased risk of death.

## Methods

### Study setting

The study was conducted at Kilifi County Hospital (KCH), located in a rural area on the Kenyan coast. The community of approximately 262,000 residents in an area of 891 km^2^ surrounding the hospital is enumerated by the Kilifi Health and Demographic Surveillance System (KHDSS) every 4 months [11]. The antenatal HIV prevalence is 4.9% [11], and KCH provides care for HIV-exposed and infected children. Services are available for both inpatient and outpatient care for acute malnutrition. KCH serves patients living within and outside the KHDSS area.

### Study population

Children aged 2 to 59 months resident within the KHDSS who were admitted to KCH between January 2007 and December 2015 were eligible for inclusion. Children discharged alive and followed up in KHDSS census rounds until August 2017 were eligible for analysis of post-discharge mortality.

### Study design

We conducted a retrospective cohort study. The outcomes of interest were death in the hospital and during the 1 year after discharge. Exposures examined were demographic, clinical, nutritional, and biochemical characteristics at hospitalization.

### Clinical procedures

Clinical history and examination, anthropometry, blood slide for malaria, and full blood count were systematically undertaken at admission for acute pediatric patients and entered onto an electronic database linked to the KHDSS. HIV testing using two rapid antibody tests, Determine (Inverness Medical, FL, USA) and Unigold (Trinity Biotech, Bray, Ireland), was offered to all pediatric admissions according to national guidelines [12]. Families of patients with a positive test were counseled and referred for comprehensive care. Blood culture was systematically undertaken at admission by methods previously published [13]. Biochemistry and blood gas tests were performed on children with danger signs at physician discretion.

Trained clinical assistants measured mid-upper arm circumference (MUAC) (TALC, St Albans, UK), weight with an electronic scale (Seca, Birmingham, UK) that was checked for consistency weekly, and length using a measuring board of standard UNICEF design (for children younger than 2 years or those who could not stand) or height using a stadiometer (Seca, Birmingham, UK). Inpatient management of severe acute malnutrition (SAM) was based on one or more of weight-for-length *z* score, MUAC, or the presence of kwashiorkor, and followed WHO guidelines. Children with SAM were discharged to a therapeutic and/or supplementary feeding program as per national guidelines.

Inpatient management followed WHO guidelines; children with diarrheal disease received rehydration as required and oral zinc for 10 days. Antibiotics were prescribed for bloody diarrhea [14].

### Definitions

Diarrhea was defined by WHO criteria (2005): the passage of unusually loose or watery stools, at least three times in a 24-h period [15]. Persistent diarrhea was defined as diarrhea lasting at least 14 days. Dysentery was defined as observation of blood in stools during acute diarrhea by parents or physicians. “Some dehydration” was defined as the presence of two or more signs from as follows: restless, irritable condition; sunken eyes; thirsty, drinks eagerly; and skin turgor: skin pinch goes back slowly. “Severe dehydration” was defined as the presence of two or more signs from as follows: lethargic or unconscious condition, sunken eyes, drinks poorly or unable to drink, and skin pinch returns very slowly. The numbers of children with severe dehydration were reported separately from those with some dehydration. Temperature gradient was detected by the clinician running their hand down the patient’s arm or leg and defined as reduced temperature in distal compared to proximal limbs. Shock was defined as the presence of at least one sign of weak/absent peripheral pulse, conscious level less than alert, cold hands and temperature gradient, or capillary refill time > 3 s. Impaired consciousness was defined as “prostration” (inability to sit unassisted (≥ 1 year), inability to drink or breast feed (< 1 year)) or “coma” (Blantyre coma score ≤ 2). Severe pneumonia was defined using the WHO 2013 syndromic criteria as cough or difficulty breathing plus either lower chest wall indrawing or inability to breastfeed/drink/vomiting everything, impaired consciousness, central cyanosis, or peripheral oxygen saturation < 90% by pulse oximetry [14]. Hypothermia was defined as axillary temperature < 36 °C, and fever as axillary temperature > 37.5 °C. Clinical signs at admission were entered directly into a database by trained clinicians who provided care according to the WHO guidelines [14]. Severe anemia was defined as hemoglobin < 5 g/dl. An abnormal white blood cell count was defined as < 4 or > 12 × 10^9^/L. Biochemical definitions are given in Additional file 1: Table S4.

### Ethical considerations

The study was approved by the Kenya Medical Research Institute (KEMRI) National Ethical Review Committee (SCC 2778). Informed consent was given in writing by parents/guardians for their child’s participation in the study.

### Statistical analysis

The primary analysis included all clinical, anthropometric, and the biochemical parameters that were systematically measured at admission (HIV, bacteremia, malaria, hemoglobin, and CBC). Where anthropometry was categorized into groups, missing values were also analyzed as a separate category. In this analysis, SAM was defined as MUAC < 11.0 cm for children < 6 months, MUAC < 11.5 cm for children ≥ 6 months, or presence of edema at any age. Moderate acute malnutrition (MAM) was defined as MUAC 11.0 to 12.0 cm and MUAC 11.5 to 12.5 cm for children aged < 6 months and ≥ 6 months respectively. Children were considered to have no malnutrition if MUAC ≥ 12.0 cm or ≥ 12.5 cm and were aged < 6 months or ≥ 6 months respectively. *z* score for height/length for age (HAZ) was calculated using the 2006 WHO growth standards [16]. Age in months was categorized into four groups: < 6, 6–11, 12–23, and ≥ 24 months. In both inpatient and post-discharge analyses, we used MUAC and HAZ as markers of malnutrition because they are less affected by dehydration than weight-based indices [17]. To further test the performance of MUAC across different conditions, we compared its predictive value for mortality with and without diarrhea.

Amongst children with diarrhea, we examined associations of characteristics at admission with inpatient mortality using a backward stepwise log-binomial regression retaining variables with *P* < 0.1 and reported risk ratios and their respective 95% confidence intervals for variables in the final model with *P* < 0.05. We performed a sensitivity analysis to examine admission features associated with inpatient mortality amongst children admitted with diarrhea but non-KHDSS residents.

To classify the admission diagnosis, we created four groups of children, diarrhea only (excluding children with a severe pneumonia co-morbidity), diarrhea and severe pneumonia, severe pneumonia only (excluding children with a diarrhea co-morbidity), and other diagnoses (without diarrhea and without severe pneumonia), and compared the risk of both inpatient and post-discharge mortality of each group with the diarrhea only as the reference.

For the post-discharge analysis, we used the KDHSS census data from January 2007 to August 2017 linked with KCH admissions between January 2007 and December 2015. Time at risk was calculated from hospital discharge to 365 days later, or the date of out-migration or death. Where a child had multiple admissions during the study period, we used the latest admission and treated earlier admissions as a binary variable “prior admissions” because few children (106/4567 (2.3%)) had more than one prior admission. We performed a single discharge analysis, considering only the latest admission during the study period. We plotted Kaplan-Meier curves and used Cox proportional regression analysis to test associations with post-discharge mortality. We tested for interactions by comparing models with and without interaction term using likelihood-ratio *χ*^2^ tests. Survival distributions were compared using a log-rank test. Variables were investigated as potential risk factors for post-discharge mortality based on the previous work [7, 18]. We assessed the multivariable regression models’ goodness of fit using Akaike information criterion (AIC) and area under receiver operating characteristic curves (AUC).

A similar secondary analysis was done including the biochemical features that were not systematically collected. In this analysis, the missing laboratory results were classified as a separate category as they were assumed to not have been missed at random.

No formal sample size estimation was done because the data from all children admitted with diarrhea in the study period were included in the analysis. We analyzed approximately one independent variable for every 10 outcomes [19]. Statistical analyses were done using STATA 13.1 (College Station, TX, USA).

## Results

**Fig. 1:**
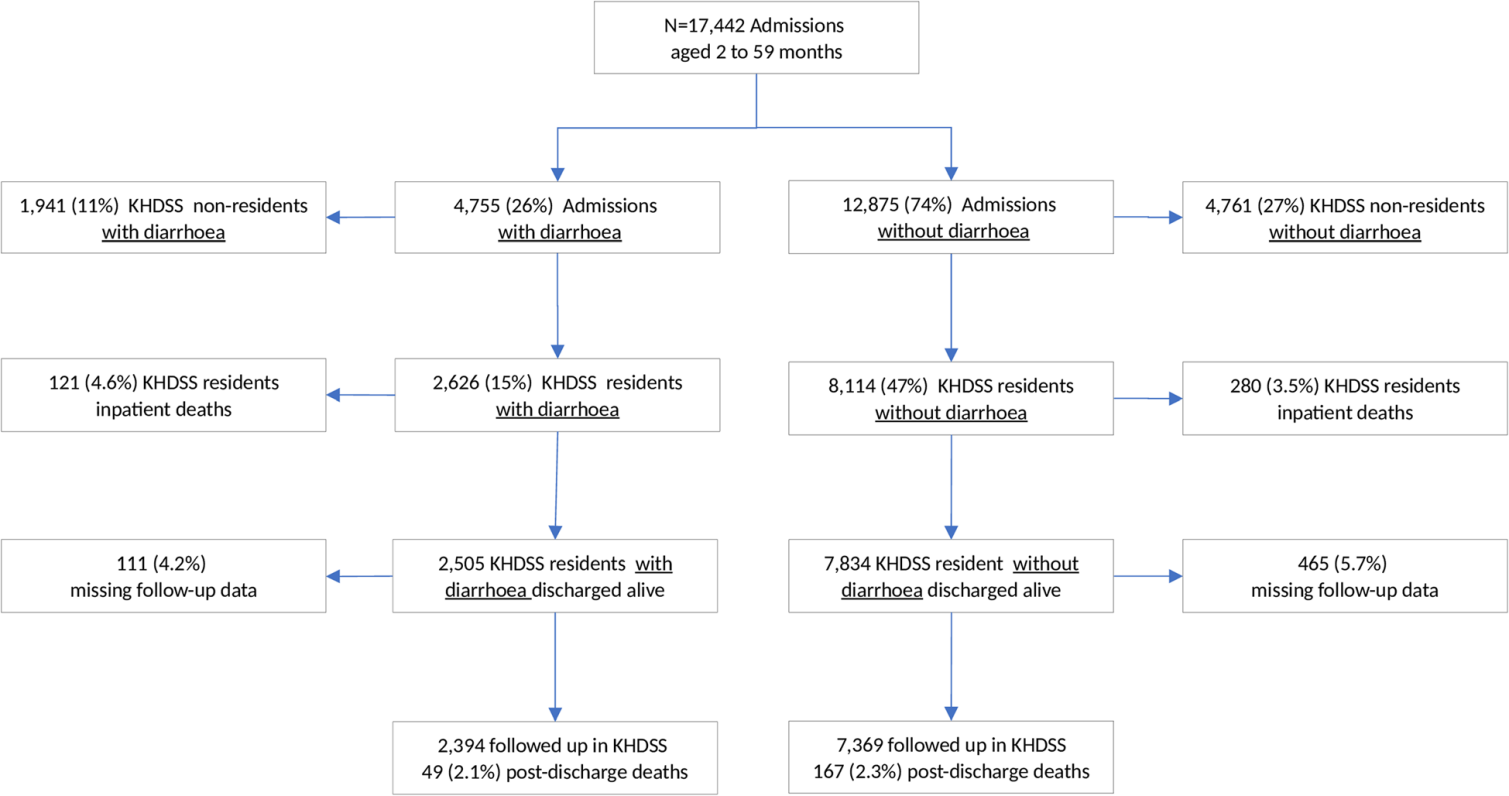
Flow diagram of recruitment and follow-up

Overall, 2626/17,442 (15%) of eligible admissions were admitted with diarrhea and were KHDSS residents (Fig. 1). Amongst these 2626 children admitted with diarrhea, 2573 (98%), 53 (2.0%), and 57 (2.2%) children had acute, persistent, and bloody diarrhea respectively. Their median age was 13 months (interquartile range (IQR) 8–21), and 1109 (42%) were female (Table 1). One hundred and thirty children (5.0%) had a positive HIV antibody test, and 415/2626 (16%) children were severely malnourished. Signs of some dehydration and severe dehydration were present in 674 (26%) and 884 (34%) children respectively. The overall median (IQR) days of hospitalization was 3 (2 to 5) amongst survivors and 2 (0 to 8) amongst those who died (*P* = 0.01.) The leading discharge diagnoses for the 2626 children admitted with diarrhea were gastroenteritis (1568 (60%)), malnutrition (339 (13%)), and lower respiratory tract infection (274 (10%)) (Additional file 1: Table S1).

**Table 1:** Study participants’ characteristics at admission

	All eligible admissions (*N* = 17,442)^a^	KHDSS resident admissions with diarrhea (*N* = 2626)	KHDSS resident admissions without diarrhea (*N* = 8114)	*P* value
Demographics				
Age in months, median (IQR)	18 (9–32)	13 (8–21)	22 (11–37)	< 0.001
Sex (female)	7518 (43)	1109 (42)	3545 (44)	0.19
Prior hospital admission	2782 (16)	347 (13)	1565 (19)	< 0.001
Clinical features				
Axillary temp < 36 °C	596 (3.4)	79 (3.0)	305 (3.8)	0.02
Axillary temp 36 to 37.5 °C	13,840 (79)	1154 (44)	3339 (42)	
Axillary temp > 37.5 °C	3006 (17)	1393 (53)	4470 (55)	
Tachypnea^b^	6368 (37)	768 (29)	3144 (39)	< 0.001
Tachycardia^c^	8255 (47)	1049 (40)	4171 (51)	< 0.001
Indrawing	4609 (26)	404 (15)	2362 (29)	< 0.001
Hypoxia (SaO2 < 90%)	907 (5.2)	75 (2.9)	393 (4.8)	< 0.001
Capillary refill > 2 s	604 (3.5)	152 (5.8)	143 (1.8)	< 0.001
Temperature gradient	1045 (6.0)	264 (10)	306 (3.8)	< 0.001
Weak pulse	496 (2.8)	151 (5.6)	92 (1.1)	< 0.001
Lethargy	2068 (12)	598 (23)	665 (8.2)	< 0.001
Sunken eyes	2552 (15)	1251 (48)	186 (2.3)	< 0.001
Reduced skin turgor	1540 (8.8)	733 (28)	91 (1.1)	< 0.001
No dehydration	12,074 (69)	1068 (41)	6568 (81)	< 0.001
Some dehydration	3466 (20)	674 (26)	1399 (17)	
Severe dehydration	1902 (11)	884 (34)	147 (1.8)	
Shock^d^	55 (0.3)	17 (0.7)	8 (0.1)	< 0.001
Impaired consciousness^e^	1737 (10)	178 (6.8)	763 (9.4)	< 0.001
Laboratory features				
HIV antibody positive	719 (4.1)	130 (5.0)	209 (2.6)	< 0.001
Malaria slide positive	2352 (13)	110 (4.2)	1431 (18)	< 0.001
Bacteremia	634 (3.6)	88 (3.4)	282 (3.5)	0.76
Severe anemia (Hb < 5 g/dL)	1.301 (7.5)	100 (3.8)	638 (7.9)	< 0.001
Leucopenia^f^ (WBC < 4 × 10^9^/L)	164 (1.0)	25 (1.0)	65 (0.8)	< 0.001
Leucocytosis (WBC > 12 × 10^9^/L)	9265 (53)	1323 (50)	4372 (54)	
Nutritional status				
Kwashiorkor	807 (4.6)	117 (4.5)	243 (3.0)	< 0.001
MUAC (cm) ± SD	13.5 ± 1.8	13.0 ± 1.6	13.9 ± 1.7	< 0.001
HAZ ± SD	− 1.4 ± 1.7	− 1.4 ± 1.7	− 1.3 ± 1.6	0.09

*KHDSS* Kilifi Health and Demographic Surveillance System, *SD* standard deviation, *MUAC* mid-upper arm circumference. ^a^Eligible admissions were children admitted from 2007 to 2015 aged between 2 and 59 months. ^b^Tachypnea was defined as respiration rate > 50 for children < 12 months and > 40 breaths/min for children ≥ 12 months. ^c^Tachycardia was defined as heart rate > 180 for children < 12 months and > 140 beats/min for children ≥ 12 months. ^d^Shock was defined as unconscious or weak pulse volume or the presence of temperature gradient or capillary refill > 3 s. ^e^Impaired consciousness level if “prostrate” or “unconscious.” ^f^Normal white blood cell range (WBC) was 4 to 12 × 10^9^/L

In contrast, the 8114/17,442 (47%) KHDSS resident children admitted without diarrhea were older (median (IQR) 22 (11–37) months, (*P* < 0.001)) and their clinical signs differed from children admitted with diarrhea (Table 1).

### Inpatient mortality

Of the 2626 KHDSS resident admissions with diarrhea, 121 (4.6%) died in the hospital (Fig. 1) and distributions of deaths did not differ by age (*P* = 0.12) (Additional file 1: Table S2). Inpatient mortality was higher in children admitted with both diarrhea and severe pneumonia (age- and sex-adjusted RR 5.13 (95% CI 3.64 to 7.23, *P* < 0.001)) and severe pneumonia only (age- and sex-adjusted RR 2.26 (95% CI 1.69 to 3.04, *P* < 0.001)) but not amongst children admitted without diarrhea or pneumonia (age- and sex-adjusted RR 0.78 (95% CI 0.57 to 1.07, *P* = 0.12)) compared to children admitted with diarrhea alone.

### Risk factors for inpatient mortality for children with diarrhea

Amongst 2626 KHDSS resident children admitted with diarrhea, inpatient mortality was associated with signs of circulatory impairment, positive HIV antibody test, bacteremia, leukocytosis, and nutritional status (Table 2). We found no evidence of interaction between HIV status and HAZ (*P* = 0.08), age (*P* = 0.15), or MUAC (*P* = 0.70) on inpatient mortality. Children with missing MUAC or HAZ had very high inpatient mortality, 11/48 (23%) and 19/125 (15%) respectively, largely because deaths occurred before they could be measured (Additional file 1: Table S3). A 1-cm increase in MUAC was associated with 36% reduction in risk of inpatient death (Table 2). However, in multivariate models, age, sex, HAZ, and prior admission to Kilifi County Hospital were not associated with inpatient mortality (Table 2). MUAC had similar predictive value for mortality amongst children admitted with and without diarrhea (AUROC 0.78 (95% CI 0.72 to 0.83) and AUROC 0.76 (95% CI 0.70 to 0.81) respectively, *P* = 0.76). The final multivariable model equation is provided in Additional file 1: Box S1.

**Table 2:** Univariable and multivariable analyses of factors associated with inpatient death amongst children admitted with diarrhea

	Deaths (*N* = 121)^a^	Univariable analysis			Multivariable analysis		
		Crude RR	95% CI	*P* value	Adjusted RR	95% CI	*P* value
Demographics							
Age (months)	–	1.00	0.98–1.01	0.98			
Sex (female)	61 (50)	1.39	0.98–1.97	0.06			
Prior hospital admission	23 (19)	1.54	0.99–2.39	0.05			
Clinical features							
Persistent diarrhea	6 (5.0)	2.53	1.17–5.49	0.02			
Bloody diarrhea	3 (2.5)	1.15	0.38–3.50	0.81			
Dehydration status							
No dehydration	22 (18)	1.0	Reference				
Some dehydration	33 (27)	2.38	1.40–4.04	0.001			
Severe dehydration	66 (55)	3.62	2.26–5.82	< 0.001			
Tachypnea^b^	62 (51)	2.58	1.82–3.66	< 0.001	1.98	1.34–2.93	0.001
Tachycardia^c^	43 (36)	0.86	0.60–1.24	0.42			
Lower chest wall indrawing	46 (38)	3.37	2.37–4.79	< 0.001			
Hypoxia (SaO2 < 90%)	25 (21)	8.86	6.09–12.89	< 0.001			
Capillary refill > 2 s	48 (40)	10.70	7.73–14.82	< 0.001	2.31	1.35–3.95	0.002
Temperature gradient	56 (46)	7.71	5.52–10.76	< 0.001			
Weak pulse	47 (39)	10.41	7.51–14.43	< 0.001			
Impaired consciousness^d^	53 (44)	10.72	7.74–14.84	< 0.001	3.29	195–5.54	< 0.001
Systematic laboratory test features							
HIV antibody positive	24 (20)	5.59	3. 65–8.58	< 0.001	2.40	1.44–3.99	0.001
Bacteremia	24 (20)	7.14	4.82–10.57	< 0.001	2.05	1.18–3.57	0.01
Malaria slide positive	2 (1.7)	0.43	0. 11–1.71	0.23			
Severe anemia (Hb < 5 g/dL)	16 (13)	3.98	2.43–6.50	< 0.001			
Leucopenia^e^ (WBC < 4 × 10^9^/L)	8 (6.6)	13.93	6.98–27.78	< 0.001			
Leucocytosis (WBC > 12 × 10^9^/L)	77 (64)	2.53	1. 62–3.95	< 0.001	2.24	1.41–3.58	0.001
Nutritional status							
Kwashiorkor	19 (16)	3.99	2.54–6.29	< 0.001			
MUAC per centimeter	–	0.61	0.59–0.64	< 0.001	0.64	0.57–0.71	< 0.001
Height-for-age *z* score	–	0.65	0.59–0.73	< 0.001			
Model performance							
AUC (95% CI)					0.89 (086–0.93)		
AIC					1655.5		

Variables’ missing results in the multivariable model were dropped using the stepwise approach. *AUC* area under receiver operating characteristics, *AIC* Akaike information criterion. ^a^Number of deaths and proportion of deaths. ^b^Tachypnea was defined as respiration rate > 50 for children < 12 months and > 40 breaths/min for children ≥ 12 months. ^c^Tachycardia was defined as heart rate > 180 for children < 12 months and > 140 beats/min for children ≥ 12 months. ^d^Impaired consciousness if “prostrate” or “unconscious.” ^e^Normal white blood cells (WBC) were 4 to 12 × 10^9^/L

In the secondary analysis including biochemical features at admission, hyponatremia, hyperkalemia, and hyperglycemia were all associated with increased inpatient mortality (Additional file 1: Table S4).

### Post-discharge mortality

Of the 2505 children admitted with diarrhea, who were discharged alive, 2394 (96%) were followed up for 1 year post-discharge, giving 2295 child-years of observation (cyo), during which 49 (2.1%) children died (Fig. 1). The post-discharge mortality rate was 21 (95% CI 16–28) deaths per 1000 cyo and did not differ across the age groups (*P* = 0.54) (Additional file 1: Table S2). Of the 49 post-discharge deaths, only 2/49 (4.1%) deaths occurred during subsequent re-admission at KCH. Twenty-six (53%) deaths occurred within the first 3 months, during 177 cyo, 147 deaths (95% CI 100–216) per 1000 cyo. Overall, of the 170 deaths during admission and post-discharge, amongst children admitted with diarrhea, 49 (29%) occurred after the discharge.

**Fig. 2:**
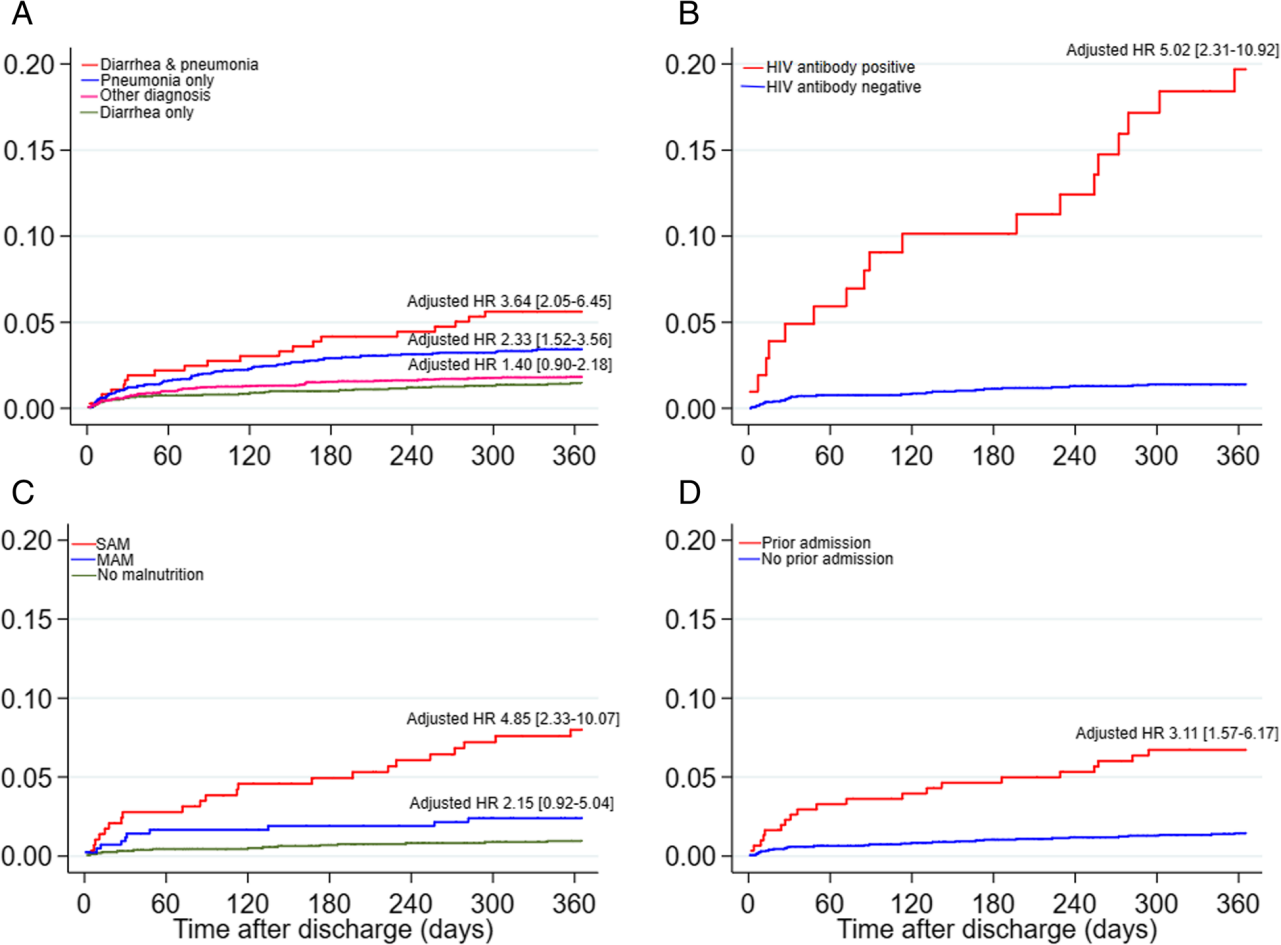
Nelson-Aalen plots of cumulative hazard of post-discharge mortality by **a** admission diagnosis, **b** HIV antibody status, **c** nutrition status, and **d** prior hospital admission

Compared to children admitted with diarrhea alone and discharged alive, the hazard of post-discharge mortality was not significantly different amongst admissions without diarrhea or severe pneumonia (age- and sex-adjusted hazard ratio 1.40 (95% CI 0.90 to 2.18), *P* = 0.13) (Fig. 2a and Additional file 1: Table S5). However, admissions with both diarrhea and severe pneumonia or severe pneumonia alone had significantly higher hazards of post-discharge mortality (age- and sex-adjusted hazard ratio 3.64 (95% CI 2.05 to 6.45), *P* < 0.001; 2.33 (95% CI 1.52 to 3.56), *P* < 0.001 respectively) compared to admissions with diarrhea only (Fig. 2a and Additional file 1: Table S5).

### Risk factors for post-discharge mortality after admission with diarrhea

Amongst children admitted to KCH with diarrhea, post-discharge mortality was associated with prior hospital admission, admission with lower chest wall indrawing, positive HIV antibody test, bacteremia, and nutritional status (Table 3, Fig. 2b–d, and Additional file 1: Table S6). There was no evidence of interaction between HIV status and age (*P* = 0.54), MUAC (*P* = 0.12), or HAZ (*P* = 0.96) on post-discharge mortality. The final post-discharge multivariable model equation is provided in Additional file 1: Box S1 and Additional file 1: Figure S1. In the secondary analysis, no biochemical features were associated with post-discharge mortality (Additional file 1: Table S7).

**Table 3:** Univariable and multivariable analyses of factors associated with post-discharge deaths amongst children admitted with diarrhea and residents of KHDSS

	Deaths (*N* = 49)^a^	Univariable analysis			Multivariable analysis		
		Crude HR	95% CI	*P* value	Adjusted HR	95% CI	*P* value
Demographic features							
Age (months)	–	1.00	0.97–1.02	0.80			
Sex (female)	22 (45)	1.12	0.64–1.96	0.70			
Prior hospital admission	20 (41)	4.71	2.66–8.32	< 0.001	3.11	1.57–6.17	0.001
Clinical features							
Persistent diarrhea	3 (6.1)	3.51	1.11–11.13	0.03			
Bloody diarrhea	1 (2.0)	0.90	0.12–6.46	0.92			
Dehydration status							
No dehydration	16 (33)	1.0	Reference				
Some dehydration	9 (18)	0.90	0.40–2.04	0.80			
Severe dehydration	24 (49)	1.92	1.02–3.61	0.04			
Tachypnea^b^	18 (37)	1.50	0.83–2.68	0.18			
Tachycardia^c^	19 (39)	0.95	0.53–1.68	0.85			
Lower chest wall indrawing	16 (33)	2.90	1.59–5.26	< 0.001	2.00	1.03–3.79	0.04
Hypoxia (SaO2 < 90%)	2 (4.1)	2.03	0.49–8.34	0.33			
Capillary refill > 2 s	6 (12)	3.16	1.35–7.43	0.008			
Temperature gradient	7 (14)	1.85	0.83–4.12	0.13			
Weak pulse	5 (10)	2.50	1.00–6.30	0.05			
Impaired consciousness^d^	6 (12)	2.75	1.17–6.45	0.02			
Systematic lab test features							
HIV antibody positive	18 (37)	13.76	7.60–24.91	< 0.001	5.02	2.31–10.92	< 0.001
Bacteremia	6 (12)	5.41	2.27–12.89	< 0.001	3.69	1.64–10.14	0.01
Malaria slide positive	3 (6.1)	1.52	0.47–4.93	0.48			
Severe anemia (Hb < 5 g/dL)	6 (12)	4.20	1.78–9.90	0.001			
Leucopenia^e^ (WBC < 4 × 10^9^/L)	1 (2.0)	4.07	0.52–32.09	0.18			
Leucocytosis (WBC > 12 × 10^9^/L)	30 (61)	1.74	0.94–3.23	0.08			
Nutritional status							
Kwashiorkor	4 (8.2)	2.21	0.80–6.11	0.13			
MUAC per centimeter		0.55	0.47–0.64	< 0.001	0.67	0.56–0.81	< 0.001
Height-for-age *z* score		0.62	0.52–0.73	< 0.001			
Model performance							
AUC (95% CI)					0.87 (0.81–0.94)		
AIC					637.9		

Variables’ missing results in the multivariable model were dropped using the stepwise approach. *AUC* area under receiver operating characteristics, *AIC* Akaike information criterion. ^a^Number of deaths and proportion of post-discharge deaths. ^b^Tachypnea was defined as respiration rate > 50 for children < 12 months and > 40 breaths/min for children ≥ 12 months. ^c^Tachycardia was defined as heart rate > 180 for children< 12 months and > 140 beats/min for children ≥ 12 months. ^d^Impaired consciousness if “prostrate” or “unconscious.” ^e^Normal white blood cell (WBC) count was 4 to 12 × 10^9^/L

## Discussion

Amongst KHDSS resident children admitted to the hospital with diarrhea alone, the risk of inpatient and post-discharge mortality was not significantly different from those with other diagnoses excluding severe pneumonia. Diarrhea with concomitant severe pneumonia was associated with increased inpatient mortality and post-discharge mortality compared to admission with diarrhea alone. Signs of severe infection, circulatory impairment, and biochemical disturbance at admission were, unsurprisingly, associated with inpatient mortality. As previously reported in the literature, we found hyperglycemia which is a stress response common in critically ill children to be a risk factor for increased mortality during admission [20]. In contrast, the main features associated with post-discharge mortality amongst children with diarrhea were prior hospital admission, lower chest wall indrawing, bacteremia, HIV status, and undernutrition, despite the availability of follow-up care services for the latter two.

The proportion of pediatric admissions with diarrhea in our study (15%) was lower than that amongst Tanzanian (27%) children, and the proportion with diarrhea with dehydration amongst Kenyan children at 13 hospitals was ~ 33% [21, 22]. The inpatient mortality of 4.6% in this study was similar to that in Tanzania (4.6%) and lower than that in rural western Kenya (9%), Calcutta (14%), or Haiti (13%) [21, 23–25].

The 2.1% 1-year post-discharge mortality in this study is lower than the range of three studies in Bangladesh (2.8 to 7.5%), despite their follow-up being shorter (4 and 3 months respectively) [2, 3, 5]. The Bangladesh studies were carried out before the year 2000, and it may be that hospital usage patterns have changed since then, with children presenting for admission less severely ill than previously. The lower post-discharge mortality in our cohort could be a reflection of the global decline in child mortality, associated with reduction in malaria transmission, introduction of Hib (*Haemophilus influenzae* type B) and pneumococcal vaccines, and the changing landscape of diarrheal disease since the introduction of a rotavirus vaccine [26, 27]. The rotavirus vaccine was incorporated into the Kenyan national immunization program in July 2014. The overall 12-month post-discharge mortality rate in this study of diarrhea was much lower than that previously reported amongst children treated for severe pneumonia in the same population: 21 (95% CI 16–28) vs. 32 (95% CI 26–41) deaths per 1000 cyo, implying greater persisting vulnerability indicated by an episode of pneumonia [18].

Although children aged < 6 months typically have higher mortality risks, age was not a risk factor for either inpatient or post-discharge mortality amongst children admitted with diarrhea [18, 28]. The finding that prior admissions were associated with post-discharge mortality concurs with previous reports and suggests that returning to the hospital could be a marker of incompletely treated severe illness or ongoing vulnerability. Like previous studies, undernutrition and HIV infection were the main features associated with both inpatient and post-discharge mortality [18], even though children admitted with SAM and HIV infection are usually linked to outpatient management of these conditions upon discharge. Loss to follow-up of children enrolled at HIV clinics has been estimated at 14% in East Africa after 18 months on antiretroviral treatment, and suggestions for improving retention include treatment at smaller decentralized clinics, integrating visits with those of other family members, and assisting with transport costs [29].

Strengths of our study were the systematic collection of detailed data at hospital admission and large number of children followed up in the KHDSS for more than 1 year after the hospital discharge. However, the number of outcome events was a limiting factor in the analysis, reducing the number of independent variables that could be examined. Hence, a backward stepwise method of analysis was used to eliminate variables that were not statistically significant. The post-discharge multivariable model predicting mortality requires external validation to test its generalizability. Another limitation of this study is that it is from a single hospital and does not include data on the underlying pathogens causing diarrhea. We did not analyze birthweight and gestational age as risk factors because many deliveries occurred at home and accurate data was not available. The fact that biochemical features were not systematically collected introduced bias, and so these factors could only be included in a secondary analysis.

Research to reduce excess mortality after discharge should focus on targeting care to the highest risk children, improving methods of identification of early warning indicators of subsequent deterioration, access to treatment, improving retention and outcomes of malnutrition and HIV services, and identifying other basic mechanisms amenable to intervention. Currently, the CHAIN network cohort study is examining biomedical and social mechanisms involved in post-discharge mortality [30]. We anticipate that besides making the best use of existing screening and use of services for recognized risks (malnutrition, HIV, and sickle cell disease for example), effective intervention solutions are likely to require a much better basic understanding of factors such as incompletely treated infections; pathogens acquired in the hospital; immune recovery; whether therapeutic feeding products address immunologically relevant nutrient deficiencies; intestinal dysfunction including bacterial translocation, inflammation, and malabsorption; and critical social limitations to benefitting from biomedical interventions such as extreme poverty, lack of access to care, and agency and maternal physical and mental health.

## Conclusions

We observed no difference in inpatient and post-discharge mortality between diarrhea and other diagnoses excluding severe pneumonia. Most clinical signs of illness severity were not associated with post-discharge mortality in this analysis, but children with a history of previous hospital admission, concurrent lower chest wall indrawing, SAM, bacteremia, or a positive HIV antibody test require further attention after discharge to prevent excess deaths. Clinicians should be aware of post-discharge mortality and its risk factors in order to advise parents to have a low threshold for seeking help in case of further problems. In addition, national programs may wish to target highly vulnerable children for facilitated post-discharge follow-up and care.

## Additional file

Additional file 1: Search terms for papers on post-discharge mortality in children following diarrhea admission. **Table S1.** Discharge diagnosis of children admitted with diarrhea. **Table S2.** Distribution of inpatient and post-discharge mortality by age groups amongst children admitted with diarrhea. **Table S3.** Association between nutritional status and inpatient mortality amongst children admitted with diarrhea. **Table S4.** Univariable and multivariable analysis of laboratory variables, not systematically tested, associated with inpatient deaths amongst children admitted with diarrhea. **Table S5.** Post-discharge mortality amongst children discharged alive and residents of KHDSS by admission diagnosis. **Table S6.** Association between nutritional status and post-discharge mortality amongst children admitted with diarrhea and residents of KHDSS. **Table S7.** Univariable and multivariable analysis of laboratory variables, not tested systematically, associated with post-discharge mortality amongst children admitted with diarrhea and residents of KHDSS. **Box S1.** Models for inpatient and post-discharge mortality prediction. **Figure S1.** Kernel-smoothed baseline hazard of the Post-discharge deaths model. (DOCX 119 kb)

## Abbreviations

AUROC: Area under receiver operating curve
CBC: Complete blood count
CI: Confidence intervals
cyo: Child-years of observation
GEMS: Global Enteric Multicenter Study
HAZ: Height/length-for-age *z* score
HIV: Human immunodeficiency virus
IQR: Interquartile range
KCH: Kilifi County Hospital
KEMRI: Kenya Medical Research Institute
KHDSS: Kilifi Health and Demographic Surveillance System
MAM: Moderate acute malnutrition
MUAC: Mid-upper arm circumference
OR: Odds ratio
RR: Relative risk
SAM: Severe acute malnutrition
SCC: Scientific Coordinating Committee
UNICEF: United Nations Children’s Fund
WHO: World Health Organization
