# Supplementary material for: Mortality after inpatient treatment for diarrhea in children: a cohort study
Source: BMC Med. 2019 Jan 28;17:20. doi: 10.1186/s12916-019-1258-0 (PMC6348640; doi:10.1186/s12916-019-1258-0)
Supplement: Supplementary file 1 — Search terms for papers on post-discharge mortality in children following diarrhea admission. Table S1. Discharge diagnosis of children admitted with diarrhea. Table S2. Distribution of inpatient and post-discharge mortality by age groups amongst children admitted with diarrhea. Table S3. Association between nutritional status and inpatient mortality amongst children admitted with diarrhea. Table S4. Univariable and multivariable analysis of laboratory variables, not systematically tested, associated with inpatient deaths amongst children admitted with diarrhea. Table S5. Post-discharge mortality amongst children discharged alive and residents of KHDSS by admission diagnosis. Table S6. Association between nutritional status and post-discharge mortality amongst children admitted with diarrhea and residents of KHDSS. Table S7. Univariable and multivariable analysis of laboratory variables, not tested systematically, associated with post-discharge mortality amongst children admitted with diarrhea and residents of KHDSS. Box S1. Models for inpatient and post-discharge mortality prediction. Figure S1. Kernel-smoothed baseline hazard of the Post-discharge deaths model. (DOCX 119 kb) [file 12916_2019_1258_MOESM1_ESM.docx]

**Combined additional file material**

1. Additional file S1 Search terms for papers on post-discharge mortality in children following diarrhea admission
2. Additional file Table S1**:**  Discharge diagnosis of children admitted with diarrhea.
3. Additional file Table S2: Distribution of inpatient and post-discharge mortality by age groups amongst children admitted with diarrhea.
4. Additional file Table S3: Association between nutritional status and inpatient mortality amongst children admitted with diarrhea.
5. Additional file Table S4: Univariable and multivariable analysis of laboratory variables, not systematically tested, associated with inpatient deaths amongst children admitted with diarrhea.
6. Additional file Table S5: Post-discharge mortality amongst children discharged alive and residents of KHDSS by admission diagnosis.
7. Additional file Table S6: Association between nutritional status and post-discharge mortality amongst children admitted with diarrhea and residents of KHDSS.
8. Additional file Table S7: Univariable and multivariable analysis of laboratory variables, not tested systematically, associated with post-discharge mortality amongst children admitted with diarrhea and residents of KHDSS.
9. Additional file Box S1: Models for inpatient and post-discharge mortality prediction.
10. Additional file Figure S1: Kernel-smoothed baseline hazard of the Post-discharge deaths model**.**

**Additional file 1: Search terms for papers on post-discharge mortality in children following diarrhea admission**

We conducted a search of the MEDLINE database from November 2012 to end of May 2018 using the terms ("post-discharge") OR "after-discharge") OR "post-hospital discharge") OR "after hospital discharge") OR "follow-up studies") OR "hospitalization") OR "hospitalisation") OR "longitudinal-studies")) AND ((("Child"[Mesh]) OR "Child") OR "Children")) AND ((("mortality" [Subheading]) OR "mortality") OR "Mortality"[Mesh])) AND ((("Diarrhoea"[Mesh]) OR "Diarrhoea") OR "Diarrhoea")) AND (("developing countries") OR (("Africa"[Mesh]) OR "Asia"[Mesh])))

Table S1: **Discharge diagnosis of children admitted with diarrhea.**

| **Discharge diagnosis (N=2,626)** | **Diagnosis one*** | | **Diagnosis two*** | |
| --- | --- | --- | --- | --- |
|  | All children-N (%) | Deaths (N=121)-N (%) | All children-N (%) | Deaths (N=121)-N (%) |
| Gastroenteritis | 1,568 (60) | 25 (21) | 345 (13) | 27 (22) |
| Malnutrition | 339 (13) | 47 (39) | 71 (2.7) | 10 (8.3) |
| LRTI | 274 (10) | 13 (11) | 142 (5,4) | 10 (8.3) |
| Malaria | 106 (4.0) | 1 (0.8) | 13 (0.5) | 0 |
| Febrile convulsions | 62 (2.4) | 1 (0.8) | 85 (3.2) | 1 (0.8) |
| Dysentery | 54 (2.1) | 1 (0.8) | 20 (0.8) | 0 |
| Septicaemia/sepsis | 29 (1.1) | 11 (9.1) | 33 (1.3) | 11 (9.1) |
| URTI | 27 (1.0) | 0 | 86 (3.3) | 0 |
| Others | 167 (6.4)^a^ | 22 (18)^b^ | 1831 (70)^c^ | 62 (51)^d^ |
| * Clinicians are asked to record up to 2 diagnoses at death or discharge  ^a^unclassified disease-22, accident-1, acute abdomen obstruction-7, acute flaccid paralysis-2, anaemia-15, asthma-1, bronchiolitis-10, burns-6, cellulitis/abscess-5, cerebral palsy-1, chickenpox-2, cholera-6, congenital abnormality-5, conjunctivitis-1, dehydration-6, elective surgery-3, encephalopathy-10, epilepsy-2, immunosuppression-17, measles-3, meningitis-10, neonatal sepsis-1, nephrotic syndrome-2, osteomyelitis-1,skin disease-4, poisoning-11, TB-3, Sickle cell disease-2, trauma-1, Urinary tract infection-5, viral hepatitis-2.  ^b^anaemia-1, congenital abnormality-3, encephalopathy-3, immunosuppression-7, meningitis-3, neonatal sepsis-1, TB-2, renal failure-1 and sickle cell disease-1.  ^c^TB-3, accidents-1, acute abdomen obstruction-1, anaemia-62, astha-3, bronchiolitis-8, burns-1, cellulitis/abscess-6, cerebral palsy-4, chickenpox-1, cholera-3, congenital abnormality-14, conjunctivitis-3, dehydration-48, developmental delay-1, encephalopathy-6, epilepsy-3, immunosuppression-68, measles-2, meningitis-3, nephrotic syndrome-1, skin disease-12, poisoning-5, rash-1, scabies-2, sickle cell disease-6, trauma-1, urinary tract infection-10, Viral hepatitis-3 and no secondary diagnosis-1549.  ^d^ immunosuppression-16, anaemia-6, dehydration-5, congenital abnormality-3, nephrotic syndrome-1, sickle cell disease-1, urinary tract infection-1, viral hepatitis-1, acute abdomen obstruction-1, unreported-27 | | | | |

Table S2: **Distribution of inpatient and post-discharge mortality by age groups amongst children admitted with diarrhea.**

| **Age groups** | **Inpatient mortality** | | | **Post-discharge mortality** | | |
| --- | --- | --- | --- | --- | --- | --- |
|  | **N (N=121)** | **%** | **P-value^a^** | **N (%)**  **(n=49)** | **Mortality rate (95% CI) per 1,000 cyo** | **P-value^b^** |
| <6months | 21 | 7.0 | 0.12 | 8 (1.7) | 30.5 (15.2-60.9) | 0.54 |
| 6-11 months | 32 | 3.7 |  | 19 (2.3) | 18.1 (10.7-30.6) |  |
| 12-23 months | 42 | 4.5 |  | 14 (1.7) | 23.5 (15.0-36.9) |  |
| ≥24 months | 26 | 4.9 |  | 8 (2.9) | 17.7 (8.9-35.4) |  |
| Total | 121 | 4.6 |  | 49 (2.1) | 21.4 (16.1-28.3) |  |
| ^a^-chi-square p-values, ^b^-Mantel-Haenszel test of trend p-value, cyo-Child-years observation | | | | | | |

Table S3: **Association between nutritional status and inpatient mortality amongst children admitted with diarrhea.**

|  | N=2,626 | Died (N=121)) | % died | Crude RR | Crude 95% CI | Adjusted^d^ RR | Adjusted^d^ 95% CI |
| --- | --- | --- | --- | --- | --- | --- | --- |
| Nutritional status |  |  |  |  |  |  |  |
| No malnutrition | 1,736 | 31 | 1.8 | Reference | Reference | Reference | Reference |
| MAM^a^ | 427 | 11 | 2.6 | 1.44 | 0.73-2.85 | 1.45 | 0.73-2.87 |
| SAM^b^ | 415 | 68 | 16 | 9.18 | 6.08-13.84 | 7.88 | 5.13-12.11 |
| MUAC^c^ not collected | 48 | 11 | 23 | 12.83 | 6.87-23.98 | 12.63 | 6.84-23.33 |
| Length/height for age Z-score |  |  |  |  |  |  |  |
| ≥-2 | 1,687 | 39 | 2.3 | Reference | Reference | Reference | Reference |
| -2 to -3 | 416 | 15 | 3.6 | 1.56 | 0.87-2.80 | 1.58 | 0.88-2.86 |
| <-3 | 398 | 48 | 12 | 5.22 | 3.47-7.85 | 4.50 | 2.93-6.91 |
| HAZ not collected | 125 | 19 | 15 | 6.57 | 3. 92-11.03 | 6.78 | 4.07-11.27 |
| ^a^-MAM-Moderate acute malnutrition, ^b^-SAM-severe acute malnutrition, ^c^-MUAC: Mid-upper arm circumference, ^d^-Adjusted for age, sex and HIV status, RR-Risk ratios generated using log-binomial regression models | | | | | | | |

|  | N | Deaths (N=121)^a^ | Univariable analysis | | | Multivariable analysis | | |
| --- | --- | --- | --- | --- | --- | --- | --- | --- |
|  |  |  | Crude RR | 95% CI | P-value | Adjusted RR^b^ | 95% CI | P-value |
| Laboratory variables |  |  |  |  |  |  |  |  |
| Hypernatremia (Sodium>150 mmol/L) | 21 | 1 (4.8) | 1.5 | 0.2-10.3 | 0.68 | 1.0 | 0.1-7.5 | 0.97 |
| Hyponatremia (Sodium<130 mmol/L) | 307 | 37 (12) | 3.8 | 2.5-5.7 | <0.001 | 1.8 | 1.1-2.9 | 0.02 |
| Sodium not tested | 661 | 31 (4.7) | 1.5 | 0.9-2.3 | 0.08 | 0.8 | 0.3-2.4 | 0.67 |
| Hyperkalemia (Potassium >5.5 mmol/L) | 23 | 9 (39) | 13.9 | 7.6-25.4 | <0.001 | 5.7 | 2.4-13.1 | <0.001 |
| Hypokalemia (Potassium <3.0 mmol/L) | 696 | 42 (6.0) | 2.1 | 1.4-3.3 | 0.001 | 1.1 | 0.7-1.9 | 0.60 |
| Potassium not tested | 668 | 35 (5.2) | 1.9 | 1.2 -2.9 | 0.008 | 1.8 | 0.6-5.2 | 0.31 |
| Creatinine≤80 mmol/L | 457 | 26 (5.7) | 1.0 | Reference |  |  |  |  |
| Creatinine>80 mmol/L | 95 | 12 (13) | 2.2 | 1.2-4.2 | 0.02 |  |  |  |
| Creatinine not tested | 2,074 | 83 (4.0) | 0.7 | 0.5-1.1 | 0.11 |  |  |  |
| Hyperglycemia (Blood glucose>7 mmol/L) | 145 | 26 (18) | 6.3 | 3.9-10.0 | <0.001 | 5.5 | 3.1-9.5 | <0.001 |
| Hypoglycemia (Blood glucose<3 mmol/L) | 188 | 13 (6.9) | 2.4 | 1.3-4.5 | 0.005 | 1.2 | 0.6-1.9 | 0.60 |
| Blood glucose not tested | 1,001 | 45 (4.5) | 1.6 | 1.0-2.4 | 0.04 | 1.2 | 0.7-1.9 | 0.52 |
| Base deficit ≥10 mmol/L | 125 | 5 (4.0) | 1.0 | Reference |  |  |  |  |
| Base deficit<10 mmol/L | 183 | 16 (8.7) | 2.2 | 0.8-5.8 | 0.12 |  |  |  |
| Base deficit not tested | 2,318 | 100 (4.3) | 1.1 | 0.4 -2.6 | 0.87 |  |  |  |
| Positive HIV antibody test | 130 | 24 (18) | 5.6 | 3.6-8.6 | <0.001 | 2.2 | 1.3-3.6 | 0.002 |
| Demographics |  |  |  |  |  |  |  |  |
| Age (months) | 2,626 | - | 1.00 | 0.9-1.0 | 0.98 |  |  |  |
| Sex (female) | 1,109 | 61 (5.5) | 1.39 | 0.9-2.0 | 0.06 |  |  |  |
| Nutritional status |  |  |  |  |  |  |  |  |
| Kwashiorkor | 117 | 19 (16) | 4.0 | 2.5-6.3 | <0.001 | 2.1 | 1.2-3.4 | 0.007 |
| MUAC per cm | 2,626 | - | 0.61 | 0.59-0.64 | <0.001 | 0.6 | 0.5-0.7 | <0.001 |
| Height-for-age z-score | 2,626 | - | 0.65 | 0.59-0.73 | <0.001 |  |  |  |
| ^a^-Represents number of deaths and proportion of inpatients deaths, Normal sodium level was 130 to 150 mmol/L, Normal Potassium level was 3 to 5.5 mmol/L, Normal blood glucose level was 3 to 7 mmol/L, ^b^-missing adjusted risk ratios were dropped in the step-wise multivariable model | | | | | | | | |

Table S4: **Univariable and multivariable analysis of laboratory variables, not systematically tested, associated with inpatient deaths amongst children admitted with diarrhea.**

Table S5: **Post-discharge mortality amongst children discharged alive and residents of KHDSS by admission diagnosis.**

| **Admission diagnosis** | **N** | **Post-discharge deaths** | **Post-discharge mortality rate (per 1000)** | **Adjusted Hazard ratio (95 % CI)^b^** | **P-value** |
| --- | --- | --- | --- | --- | --- |
| Diarrhea only^a^ | 2,024 | 29 (1.4) | 14.9 (10.4-21.5) | Reference |  |
| Severe pneumonia only | 2,313 | 77 (3.3) | 34.9 (27.9-43.6) | 2.33 (1.52-3.56) | <0.001 |
| Diarrhea and severe pneumonia | 370 | 20 (5.4) | 57.2 (36.9-88.6) | 3.64 (2.05-6.45) | <0.001 |
| Other diagnosis^a^ | 5,056 | 90 (1.8) | 18.5 (15.0-22.7) | 1.40 (0.90-2.18) | 0.13 |
| ^a^-Other admission diagnosis excluding admission for severe pneumonia and diarrhea, ^b^-2024 children had diarrhea only and 370 had a co-morbidity of severe pneumonia totaling 2394 children included in the diarrhea post-discharge analysis, ^c^-adjusted for age at admission and sex. | | | | | |

Table S6: **Association between nutritional status and post-discharge mortality amongst children admitted with diarrhea and residents of KHDSS.**

|  | N=2,394 | Died (N=49) | % died | Crude Hazard Ratio | Crude 95% CI | Adjusted Hazard Ratio | Adjusted 95% CI |
| --- | --- | --- | --- | --- | --- | --- | --- |
| Nutritional status |  |  |  |  |  |  |  |
| No malnutrition | 1,630 | 14 | 0.9 | 1.0 | Reference | 1.0 | Reference |
| MAM^a^ | 397 | 9 | 2.3 | 2.66 | 1.15-6.15 | 2.15 | 0.92-5.04 |
| SAM^b^ | 331 | 24 | 7.3 | 8.73 | 4.52-16.88 | 4.85 | 2.33-10.07 |
| MUAC^c^ not collected | 36 | 2 | 5.6 | 6.73 | 1.53-29.61 | 6.26 | 1.41-27.88 |
| Length/height for age Z-score |  |  |  |  |  |  |  |
| >-2 | 1,583 | 17 | 1.1 | Reference |  | Reference |  |
| -2 to -3 | 375 | 10 | 2.7 | 2.48 | 1.14-5.42 | 2.06 | 0.95-4.49 |
| <-3 | 336 | 21 | 6.3 | 5.99 | 3.16-11.37 | 4.33 | 2.27-8.25 |
| HAZ not collected | 100 | 1 | 1.0 | 0.93 | 0.12-6.98 | 0.91 | 0.12-7.10 |
| ^a^-MAM-Moderate acute malnutrition, ^b^-SAM-severe acute malnutrition, ^c^-MUAC: Mid-upper arm circumference, ^d^-Adjusted for age, sex and HIV status | | | | | | | |

Table S7: **Univariable and multivariable analysis of laboratory variables, not tested systematically, associated with post-discharge mortality amongst children admitted with diarrhea and residents of KHDSS.**

|  | N | Deaths (N=49)^a^ | Univariable analysis | | | Multivariable analysis | | |
| --- | --- | --- | --- | --- | --- | --- | --- | --- |
|  |  |  | Crude HR | 95% CI | P-value | Adjusted HR^b^ | 95% CI | P-value |
| Laboratory variables |  |  |  |  |  |  |  |  |
| Hypernatremia (Sodium>150 mmol/L) | 20 | 0 | - | - |  | - | - |  |
| Hyponatremia (Sodium<130 mmol/L) | 256 | 9 (18) | 2.5 | 1.3-5.3 | 0.02 | 2.0 | 0.9-4.4 | 0.11 |
| Sodium not tested | 600 | 18 (37) | 2.1 | 1.1-3.9 | 0.02 | 2.9 | 1.4-5.8 | 0.004 |
| Hyperkalemia (Potassium >5.5 mmol/L) | 15 | 2 (2.0) | 5.3 | 0.7-40.2 | 0.11 |  |  |  |
| Hypokalemia (Potassium <3.0 mmol/L) | 623 | 15 (30) | 1.8 | 0.9-3.5 | 0.12 |  |  |  |
| Potassium not tested | 603 | 17 (35) | 2.1 | 1.4-4.1 | 0.04 |  |  |  |
| Creatinine≤80 mmol/L | 411 | 14 (29) | 1.0 | Reference |  |  |  |  |
| Creatinine>80 mmol/L | 78 | 0 | - | - |  |  |  |  |
| Creatinine not tested | 1,905 | 35 (71) | 0.5 | 0.3-1.0 | 0.05 |  |  |  |
| Hyperglycemia (Blood glucose>7 mmol/L) | 170 | 3 (6.1) | 0.8 | 0.2-2.5 | 0.64 |  |  |  |
| Hypoglycemia (Blood glucose<3 mmol/L) | 116 | 4 (8.2) | 1.5 | 0.5-4.2 | 0.47 |  |  |  |
| Blood glucose not tested | 907 | 14 (29) | 0.7 | 0.3-1.3 | 0.20 |  |  |  |
| Base deficit ≥10 mmol/L | 112 | 3 (6.1) | 1.0 | Reference |  |  |  |  |
| Base deficit<10 mmol/L | 163 | 4 (8.2) | 0.9 | 0.2-4.1 | 0.92 |  |  |  |
| Base deficit not tested | 2,119 | 42 (86) | 0.7 | 0.2-2.4 | 0.62 |  |  |  |
| Leucopenia (wbc <4X109/L) | 18 | 1 (2.0) | 4.1 | 0.5-30.8 | 0.17 | 1.6 | 0.2-12.4 | 0.66 |
| Leucocytosis (wbc >12X109/L) | 1,180 | 30 (61) | 1.7 | 0.9-3.2 | 0.08 | 1.9 | 1.0-3.5 | 0.05 |
| Missing WBC | 173 | 3 (6.1) | 1.2 | 0.4-4.2 | 0.75 | 1.2 | 0.3-4.6 | 0.74 |
| Positive HIV antibody test | 104 | 18 (37) | 13.8 | 7.6-25.0 | <0.001 | 6.5 | 3.3-12.6 | <0.001 |
| Demographics |  |  |  |  |  |  |  |  |
| Age (months) | 2,394 | - | 1.00 | 0.97-1.02 | 0.80 |  |  |  |
| Gender (female) | 1,009 | 22 (45) | 1.12 | 0.64-1.96 | 0.70 |  |  |  |
| Nutritional status |  |  |  |  |  |  |  |  |
| Kwashiorkor | 94 | 4 (8.2) | 2.21 | 0.80-6.11 | 0.13 |  |  |  |
| MUAC per cm | 2.358 | - | 0.55 | 0.47-0.64 | <0.001 | 0.7 | 0.6-0.9 | <0.001 |
| Height-for-age z-score | 2,294 | - | 0.62 | 0.52-0.73 | <0.001 | 0.8 | 0.6-0.9 | 0.02 |
| ^a^-Represents number of deaths and proportion of inpatient deaths, Normal sodium level was 130 to 150 mmol/L, Normal Potassium level was 3 to 5.5 mmol/L, Normal blood glucose level was 3 to 7 mmol/L, ^b^-missing adjusted hazard ratios were dropped in the step-wise multivariable model | | | | | | | | |

Box S1: **Models for inpatient and post-discharge mortality prediction.**

**Inpatient deaths model coeeficients**

Logit (P) = 0.89 + (0.68; Tachypnea) + (0.84; Capillary refill >2 seconds) + (1.19; Impaired consciousness) + (0.87; HIV antibody positive) + (0.72; Bacteremia) + (0.81; Leucocytosis) + (-0.45; MUAC).

**Post-discharge deaths model coefficients**

H(t)=h_0_(t) * exp ((1.13; Prior hospital admission) + (0.68; Lower chest wall indrawing) + (1.61; HIV antibody positive) + (1.30; Bacteremia) + (-0.40; MUAC)).

h_0_(t)-is the baseline hazard which is the value of hazard when all the covariates equal to zero. This value changes over time and has been estimated using fractional polynomial (below) and plotted in Additional file Figure 1 below:

ln*H*_0_ *(t)* = (-0.04) + 0.11*t* ^0.5^ + 0.002*t*

|   Smoothed baseline hazard estimated using *stkerhaz* STATA macro. The y-axis is the time under risk (in days). The smoothed baseline hazard is estimated for three bandwidths; 30, 60 and 90 days. |
| --- |

Figure S1: **Kernel-smoothed baseline hazard of the Post-discharge deaths model.**
